# Supplementary material for: Downregulation of MMP-9 Enhances the Anti-Migratory Effect of Cyclophosphamide in MDA-MB-231 and MCF-7 Breast Cancer Cell Lines
Source: Int J Mol Sci. 2021 Nov 26;22(23):12783. doi: 10.3390/ijms222312783 (PMC8657655; doi:10.3390/ijms222312783)
Supplement: Supplementary file 1 [file ijms-22-12783-s001.zip › ijms-1472141-supplementary.pdf]

International Journal of
Molecular Sciences

MDPI

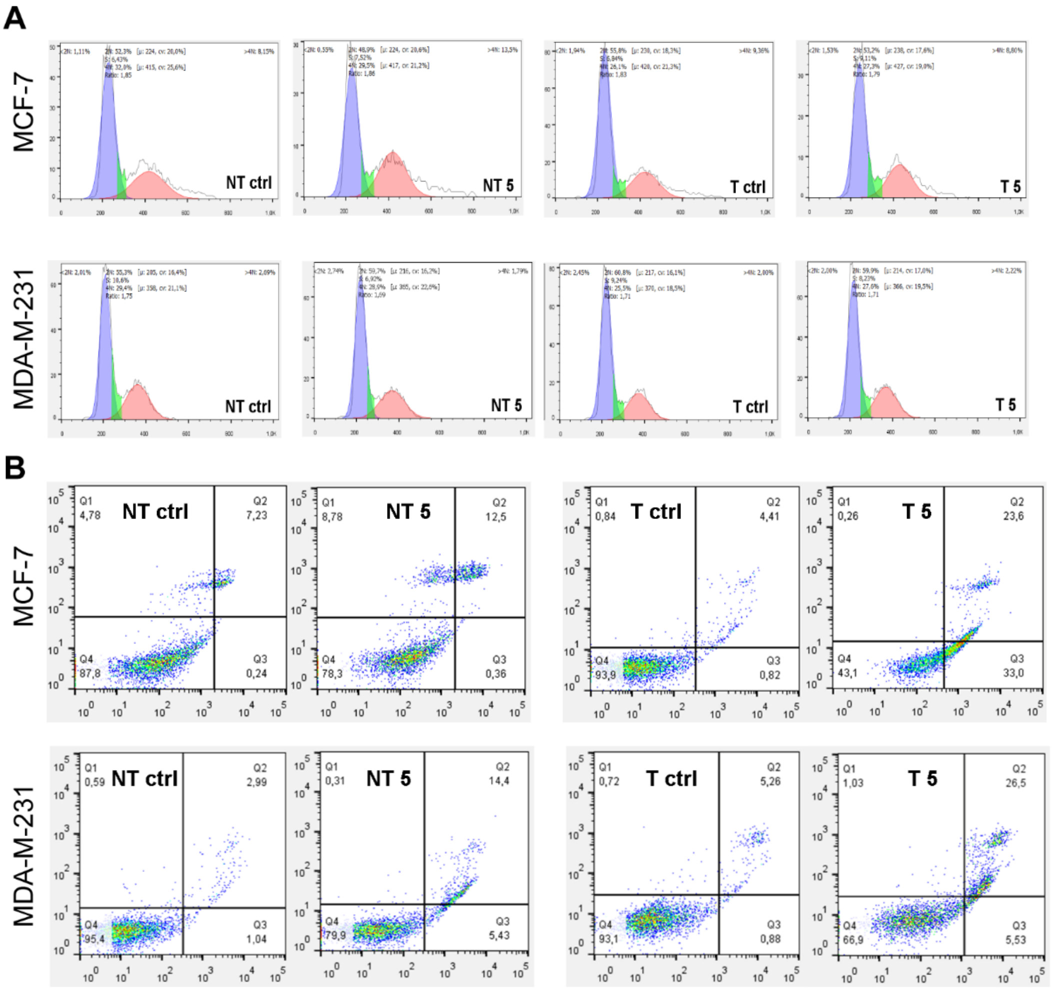

Figure S1. (**A**) The representative plots of PI staining for the cell cycle analysis. (**B**) The representative plots of Annexin V/PI staining for the apoptosis analysis. .
